# Improving Wrist Strength Assessment Reliability: A Review of Handheld Dynamometry Protocols and Their Clinical Implications

**DOI:** 10.3390/jcm14145059

**Published:** 2025-07-17

**Authors:** Diego Mazzocato, Valentina Biasol, Pasquale Arcuri, Tracy Fairplay, Fabio Vita, Donati Danilo, Davide Zanin, Paolo Boccolari, Roberto Tedeschi

**Affiliations:** 1 Independent Researcher, 40100 Bologna, Italyvalentbias@gmail.com (V.B.);; 2 Department of Biomedical and Neuromotor Sciences, Alma Mater Studiorum, University of Bologna, 40126 Bologna, Italy; pasarcuri@libero.it; 3 IRCCS Istituto Ortopedico Rizzoli, 1st Orthopaedics and Traumatology Clinic, 40136 Bologna, Italy; 4 Physical Therapy and Rehabilitation Unit, Policlinico di Modena, 41125 Modena, Italy; 5 Clinical and Experimental Medicine PhD Program, University of Modena and Reggio Emilia, 41121 Modena, Italy

**Keywords:** handheld dynamometry, wrist strength assessment, muscle strength measurement, standardized testing protocols, measurement variability

## Abstract

**Background**: Handheld dynamometry (HHD) is widely utilized for assessing muscle strength, particularly in the wrist. However, variability in measurement reliability due to differences in testing protocols poses a challenge for clinical and research applications. **Methods**: The design of this study includes a scoping review of the literature, conducted following the PRISMA-ScR checklist methodology developed by the Joanna Briggs Institute. The databases most commonly cited in review articles were consulted: EBSCO, PEDro, PubMed, Scopus, and Cochrane Library. The following MeSH terms were used: “Handheld Dynamometer”, “Wrist”, “Forearm”, “Muscle”, and “Strength”. The search strings were built using combinations of these terms. Article screening was performed by three reviewers independently, blinded to each other’s selections. **Results**: The review indicates that HHD can provide reliable measurements when standardized protocols are used. Most studies reported high intra-examiner reliability with Intraclass Correlation Coefficients (ICCs) between 0.71 and 0.90. However, inter-examiner reliability showed more variability, particularly when more than two examiners were involved. The review also highlights the importance of precise dynamometer placement and consistent patient positioning in order to reduce measurement variability. **Conclusions**: While HHD is a valuable tool for wrist strength assessment, the effectiveness of its measurements largely depends on the testing procedure’s standardization. Implementing validated standardized protocols is essential in enhancing measurement reliability and ensuring their consistent application across clinical settings. Further research is needed to firmly implement these protocols and expand their application in clinical practice.

## 1. Introduction

According to the International Classification of Function, Disability and Health (ICF), muscle strength falls under the classification of “Neuromusculoskeletal and Movement-Related Functions” and is an integral part of functional performance and individual autonomy [1]. Deficiencies in muscle strength can lead to diminished daily living activities and social participation, which underscores the importance of accurate strength assessment in clinical settings [2,3]. Traditional strength measurement methods such as Manual Muscle Testing (MMT) are frequently utilized in clinical practice to evaluate the progress of interventions and determine a patient’s capability to resume work or sports activities [4]. However, MMT often relies on subjective ordinal scales, such as the Medical Research Council (MRC) scale, which are prone to interpretation variability and may not consistently reflect true muscle strength [5,6]. This variability is exacerbated at higher strength grades, where the difference between partial and maximal resistance can be subtle yet critical. Manual Muscle Testing (MMT) is based on an ordinal scale that relies on the tester’s perception and interpretation of the MRC scale’s operational definitions. This makes the results subjective and highly susceptible to inter-rater variability. In strength grades considered “normal” (4 to 5), where resistance is applied, the subjectivity of the force applied by the examiner—whether partial (grade 4) or maximal (grade 5)—makes reproducibility problematic. More sophisticated equipment like isokinetic devices (e.g., Cybex or Biodex) provides more reliable data and is often considered the “Gold Standard” in research and sports medicine settings. Despite their accuracy, these devices are not practical for routine clinical use due to their high cost, large size, and poor portability, which limits their application even in well-equipped facilities [7]. In response to these challenges, there has been an increased interest in more portable and accessible tools such as the Handheld Dynamometer (HHD). HHDs allow for both “make” and “break” tests and have the advantage of being used in various settings, including bedside assessments. In a study by Bohannon [5,8], the make test appears to primarily assess the concentric component of muscle strength, while the break test includes an eccentric contraction phase, which is known to generate higher force values but with greater variability. However, despite their practicality, the accuracy of HHDs can still be influenced by factors such as the tester’s grip strength, the muscle’s length at the time of testing, and the specific placement of the instrument on the muscle being tested [9,10,11,12,13]. These factors can lead to significant variability in the data obtained, which complicates the interpretation of muscle strength and its functional implications [14,15,16,17,18,19,20]. This Scoping Review aimed to address these inconsistencies and gaps in the current literature by providing a comprehensive evaluation of the methods used to measure wrist muscle strength using HHDs. The specific objectives of this review were to: (a) identify and analyze the existing gamma of research and evidence regarding the dynamometry of wrist muscles using HHDs; (b) synthesize the information within a hierarchical framework of research to evaluate the reliability and validity of these methods; (c) highlight the limitations and critical issues present in the current methodologies and propose alternative testing protocols or further studies that could enhance the accuracy and reliability of wrist strength measurements. By achieving these objectives, this review was designed to aid clinicians in selecting the most appropriate and effective tools for assessing wrist muscle strength, which is crucial in diagnosing and managing conditions that impact wrist function, such as lateral elbow tendinopathy, wrist fractures, and DeQuervain’s syndrome. Moreover, the insights gained from this review could inform the development of rehabilitation programs and contribute to better clinical outcomes by providing reliable muscle strength measurements that are essential in effective treatment planning.

## 2. Methods

The present scoping review was conducted following the JBI methodology [16] for scoping reviews. In accordance with the PRISMA-ScR checklist developed by the Joanna Briggs Institute, and as per its guidelines, no formal critical appraisal of individual sources of evidence was carried out. The Preferred Reporting Items for Systematic reviews and Meta-Analyses extension for Scoping Reviews (PRISMA-ScR) [17] Checklist for reporting was used.

### 2.1. Review Question

We formulated the following research question: “What are the current methods used for measuring wrist muscle strength with handheld dynamometers?”

### 2.2. Eligibility Criteria

Studies were eligible for inclusion if they met the following Population, Concept, and Context (PCC) criteria.

**Population**: The study must involve subjects who are healthy, without age limits; studies assessing subjects with a particular illness are also included provided that there is a control group consisting of healthy subjects.

**Concept**: The intervention explored in the study must involve measurements made using a handheld dynamometer (HHD).

**Context**: This study explores the use of handheld dynamometers across diverse settings such as clinical environments, research laboratories, sports training facilities, and field applications, enabling practical assessments of wrist muscle strength in both controlled and everyday environments.

### 2.3. Exclusion Criteria

Studies that did not meet the specific PCC criteria were excluded.

### 2.4. Search Strategy

The systematic literature search was conducted from October 2023 to February 2024 across multiple electronic databases, including PubMed, Scopus, Web of Science, EBSCO, and PEDro, to ensure extensive coverage of studies on wrist muscle strength using handheld dynamometers. The search targeted literature published from January 1986 onwards, reflecting the exponential increase in the use of HHDs as highlighted by Bohannon [21]. Only studies published in English were included. Studies available only as English abstracts but published in another language were excluded. The comprehensive search strings employed were as follows: PubMed, “(handheld dynamometer) AND (wrist) AND (muscle strength)”; Scopus, “(wrist AND muscle strength) AND (dynamometry)”; Web of Science, “(handheld dynamometry) AND (wrist assessments)”; EBSCO, “(handheld dynamometer) AND (wrist muscle strength)”; and PEDro, “(muscle strength) AND (wrist) AND (handheld dynamometer)”. The review focused on studies published in English, utilizing handheld dynamometers for wrist strength assessments. An initial screening of titles and abstracts was followed by a full-text review to confirm adherence to inclusion criteria concerning methodological approach and relevance. Data extraction included study design, participant demographics, dynamometer settings, and key findings related to measurement reliability and validity. Each study was also subjected to a quality assessment to identify biases and evaluate the evidence level, enhancing the reliability of our review findings. The search results were as follows: EBSCO (39 articles), PEDro (20), PubMED (162), Scopus (54), and Grey Literature (9).

### 2.5. Study Selection

To probe the potential contributions of “Grey Literature,” several manufacturers of handheld dynamometers (HHDs) were contacted via email between January and February 2023. These companies, including Hoggan Scientific (Salt Lake city, UT, USA), JTech Medical (Midvale, UT, USA), Lafayette Instrument (Lafayette, IN, USA), Mustec (Zhengzhou, Henan, China), K-invent (Montpellier, Occitanie, France), Vald Health (Brisbane, Queensland, Australia), Activeforce (San Diego, CA, USA), and Nod (Santa Clara, CA, USA), are known for their frequent use in research protocols and their presence online. The authors of this study requested their cooperation by sharing bibliographies of articles used in the design and validation of their equipment. The responses were partial, with only some companies collaborating effectively. In addition to electronic databases, our in-depth research included reviewing all the bibliographies that were included in systematic reviews collected during our searches. From these sources, studies that met our inclusion criteria were identified and selected. No study type limitations were imposed; both primary and secondary studies were included. For managing and screening research results from databases, the open-access platform Rayyan—an AI-powered tool for systematic literature reviews—was employed. Search outcomes from various databases were saved and exported in “.csv”, “.txt”, or “.ris” formats, as permitted by each electronic database. These files were then uploaded to Rayyan to commence selection procedures, including the removal of duplicates. The screening of articles began with an analysis of “Title & Abstract,” where articles not meeting the inclusion criteria were discarded. The second level of screening involved retrieving and reviewing the full texts of studies, eliminating those that did not fully satisfy the inclusion criteria. For accessing the full versions of articles, web searches were conducted for free-full-text versions or through “Galileo Discovery”. In some cases, the authors themselves were contacted to obtain the complete articles. Articles that could not be retrieved in full or that only presented an abstract in English with the full study in another language were excluded from the selection. The review was conducted by a team of three authors, with two specializing in hand therapy and clinical practice and one an expert in research methodology. Each independently determined whether studies should be included and assigned to an evidence hierarchy. After independently extracting data, they discussed their decisions in team meetings to resolve any disagreements.

### 2.6. Data Extraction and Data Synthesis

The selected studies underwent a detailed data extraction process, where essential information such as study design, participant demographics, intervention specifics, outcomes, and measurements related to HHD use were collected using a standardized form. This standardized approach ensured consistency and facilitated a comprehensive analysis across different studies. For data synthesis, the extracted information was aggregated and analyzed to evaluate the effectiveness, reliability, and validity of using handheld dynamometers for wrist strength assessments. Studies were categorized by test methods, wrist motions tested, types of dynamometers used, and reliability metrics. Both quantitative results and qualitative aspects were synthesized to identify trends and methodological differences that impacted study outcomes. This approach provided a robust evaluation of the current literature, highlighting practical implications and guiding future research directions.

## 3. Results

**Figure 1:**
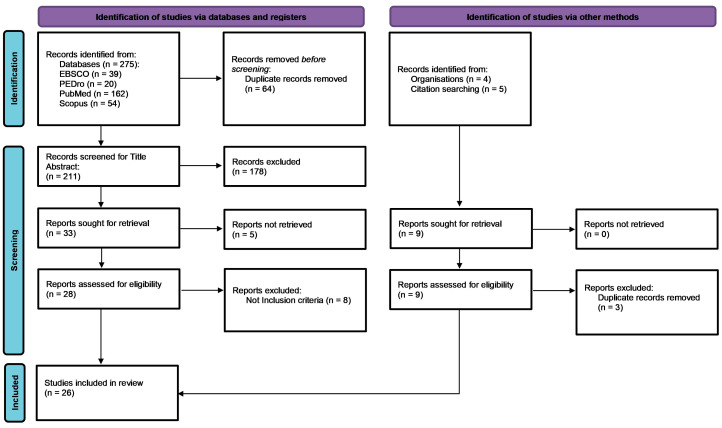
Preferred reporting items for systematic reviews and meta-analyses 2020 (PRISMA) flow diagram.

As presented in the PRISMA 2020 flow diagram (Figure 1), from 275 records identified by the initial literature searches, 275 were excluded and 26 articles were included (Table 1, Table 2, Table 3, Table 4, Table 5, Table 6, Table 7, Table 8 and Table 9). For the review work concerning the procedures found in the literature for measurements with HHD, clinical trials and comparative studies were considered. The data extracted from the selected articles were synthesized according to the previously listed objectives and are presented in (Table 1).

**Table 1:** Summary of clinical trials and comparative studies on handheld dynamometer measurement procedures.

Study	Year	Study Design	Test	HHD Model	Intra-Operator Reliability	Inter-Operator Reliability
Shimokata et al. [22]	2005	Clinical Trial	Flexion + Extension	Strain-Gauge LPR-A1KNS1	ND	ND
Wu et al. [23]	2010	Clinical Trial	Extension	Lafayette 01163	ND	ND
Nepomuceno et al. [24]	2021	Clinical Trial	Flexion + Extension	Lafayette 01165	Flex 0.91, Ext 0.89	Flex 0.85, Ext 0.84
McMahon et al. [25]	1992	Clinical Trial	Extension	SPARK	E: 0.75	E: 0.32
Kilmer et al. [26]	1997	Clinical Trial	Flexion + Extension	MicroFet	ND	F: 0.86, E: 0.87
Bohannon [8]	1995	Comparative Study	Extension	ND	ND	ND
Wadsworth, et al. [6]	1992	Clinical Trial	Extension	Chatillon	ND	ND
Rheault et al. [27]	1989	Clinical Trial	Flexion + Extension	ND	ND	F: 0.85, E: 0.91
Kimura et al. [28]	1996	Clinical Trial	Extension	Chatillon CSD500, MicroFet	E: 0.86 (Chatillon), E: 0.81 (MicroFet)	E: 0.55 (Chatillon), E: 0.52 (MicroFet)
Chou et al. [29]	2008	Clinical Trial	Extension	Microfet II	ND	ND
Nyström Eek et al. [9]	2006	Clinical Trial	Extension	Chatillon	ND	ND
Escobar et al. [30]	2017	Clinical Trial	Flexion + Extension	Lafayette 01163	ND	ND
Puharic et al. [31]	1993	Clinical Trial	Pronation + Supination	Accuforce II	Pr−Sp > 0.92	Pr−Sp > 0.90
Van der Ploeg et al. [11]	1984	Clinical Trial	Extension	Modified Wika Pressure Gauge	ND	ND
Bohannon [32]	1996	Clinical Trial	Extension	Accuforce II	ND	ND
Andrews et al. [33]	1996	Clinical Trial	Extension	Chatillon CSD400C	ND	ND
Beenakker et al. [7]	2001	Clinical Trial	Extension	CT 3001 (C.I.T. Technics)	ND	ND
Bohannon [34]	1996	Clinical Trial	Extension	Accuforce II	E: 0.95	ND
Grooten et al. [35]	2010	Clinical Trial	Extension	Nicholas Manual Muscle Tester	E: >0.90	E: 0.92
Stanković et al. [36]	2021	Clinical Trial	Flexion	Lafayette	F: 0.84	ND
Lundborg et al. [37]	2018	Clinical Trial	Flexion + Extension	Nicholas Manual Muscle Tester	ND	ND
Bohannon et al. [38]	1999	Comparative Study	Extension	(Data Not Present)	ND	ND
Karahan et al. [39]	2017	Clinical Trial	Flexion + Extension	MicroFet II	ND	ND
Toemen et al. [40]	2011	Clinical Trial	Flexion + Extension + Radial Deviation + Ulnar Deviation + Pronation + Supination	JTech Commander Power Track II	F: 0.91, E: 0.91, Pr: 0.87, Sp: 0.94, RD: 0.71, UD: 0.90	F: 0.93, E: 0.86, Pr: >0.81, Sp: >0.89, RD: >0.87, UD: >0.77
Dekkers et al. [41]	2020	Clinical Trial	Flexion + Extension	Microfet II	ND	ND
Bohannon [21]	1986	Clinical Trial	Flexion + Extension	Spark	ND	ND

Legend: E, extension; F, flexion; Pr, pronation; ND, no data; RD, radial deviation; Sp, supination; UD, ulnar deviation.

This table presents the studies in a structured format, providing clear and organized information regarding each study’s year, design, tests conducted, HHD model used, and intra- and inter-operator reliability.

## 4. Movement Directions Examined

The studies included in this review involved measuring strength during wrist movements for flexion, extension, radial deviation, ulnar deviation, pronation, and supination. Extension was the most frequently tested movement direction (24 studies), followed by flexion (11 studies). The strength expressed during other movements was examined to a lesser extent, with two studies addressing pronation–supination and a single study focusing on radial and ulnar deviation.

## 5. Test Execution Modes

It is imperative that the Standardization of Dynamometer Tests is followed in order to ensure the best test reliability and reproducibility. It is essential that both the starting position used and the test execution procedure are well defined and standardized with a precise sequenced protocol. In fact, in almost all the analyzed studies, the procedure used for evaluating all tested subjects is more or less precisely elucidated, either through a written explanation or the use of photographic support. Specifically, the key elements of the procedure include the following:

- The patient position;
- The position of the tested upper limb (shoulder, elbow, wrist);
- The position of HHD application;
- The position of stabilizing hand application;
- The type of test used.

Despite these considerations, there is currently no unified protocol for measuring wrist strength, and considerable differences are found in the test execution modes across various studies. Wrist flexion–extension studies indicated that subjects were tested both in seated and supine positions, with shoulder positioning angles ranging from 0° of abduction to 30° of abduction and elbow flexion–extension angles varying among 0°, 30°, and 90°; the wrist position was also not consistent, with tests performed in both a neutral position and at 30° of extension, while fingers were positioned in a resting state, as well as flexed or extended. The HHD application area for counterforce was always the metacarpal area, with more or less specific variations: proximal to the metacarpophalangeal joint or proximal to the head of the third metacarpal and distal metacarpal area. The direction of resistance application was always perpendicular to the segment being tested. Stabilization was applied at the forearm level while the wrist was always left off the support surface.

**Table 2:** Overview of patient positioning, testing protocol, and reliability metrics in HHD studies.

Study	Patient Position	Adjacent Segment Position	Stabilized Segment	HHD Counterforce Position	Test Type	*ICC Intra-Examiner*	*ICC Inter-Examiner*
Shimokata et al. (2014) [22]	Seated	Wrist 0°, Elbow 90°	/	/	MAKE	/	/
Nepomuceno et al. (2021) [24]	Supine	Shoulder 30° abd, Elbow 0°, Wrist 0°	/	Anterior carpal surface	MAKE	0.91	0.85
Kilmer et al. (1997) [26]	Seated	Shoulder 0°, Elbow 90°, Wrist Neutral	/	Palmar surface of hand	/	/	/
Rheault et al. (1989) [27]	Supine	Elbow 0°, Wrist 0°	Arm and forearm	Anterior metacarpal zone	BREAK	/	0.85
Escobar et al. (2017) [30]	Supine	Wrist 30° flex, Fingers ext	/	Proximal to metacarpophalangeal	BREAK	/	/
Stanković et al. (2021) [36]	Seated	Throw position	/	Proximal palm	/	0.84	/
Karahan et al. (2017) [39]	Seated	Elbow semi-flexed, Fingers flexed	Forearm fixed to support surface	Anterior surface of closed fist	/	/	/
Toemen et al. (2011) [40]	Seated	Shoulder 0°, Elbow 90°, Wrist 0°	Forearm fixed to support surface	Volar metacarpal heads	MAKE	0.91	0.93
Dekkers et al. (2020) [41]	Seated	Elbow 90°, Wrist 0°	/	/	MAKE	/	/
Bohannon et al. (1986) [21]	Seated	Shoulder 0°, Elbow 90°, Wrist Neutral	Arm and forearm	Proximal to metacarpophalangeal	MAKE	/	/

Legends /, data not available or not applicable in the specific category; abd, abduction; BREAK, type of test where the examiner applies force against the subject until the maximal resistance is overcome; ext, extension; flex, flexion; HHD, Handheld Dynamometer; ICC, Intraclass Correlation Coefficient; MAKE, type of test where the examiner holds the dynamometer static against the subject’s maximal effort.

This table summarizes the patient positioning, HHD counterforce positions, and reliability assessments (ICC values) across various studies, providing a comprehensive view of methodological approaches and outcomes in the use of handheld dynamometers for wrist strength measurements.

**Table 3:** Detailed protocols and reliability assessments for wrist strength measurement using handheld dynamometers.

Study	Patient Position	Adjacent Segment Position	Stabilized Segment	HHD Counterforce Position	Test Type	*ICC Intra-Examiner*	*ICC Inter-Examiner*
Shimokata et al. (2014) [22]	Seated	Wrist 0°, Elbow 90°	/	/	MAKE	/	/
Nepomuceno et al. (2021) [24]	Supine	Shoulder 30° abd, Elbow 0°, Wrist 0°	/	Anterior carpal surface	MAKE	0.89	0.84
McMahon et al. (1992) [25]	Seated	Wrist neutral, Fingers flex	Forearm	/	MAKE	0.75	0.32
Kilmer et al. (1997) [26]	Seated	Shoulder 0°, Elbow 90°, Wrist 0°	/	Dorsal hand surface	/	/	/
Wadsworth et al. (1992) [6]	Seated	Elbow Flexed, Wrist 15° ext	/	Proximal metacarpophalangeal	MAKE	/	/
Rheault et al. (1989) [27]	Supine	Elbow 0°, Wrist 0°	Arm and forearm	Anterior metacarpal zone	BREAK	/	0.91
Kimura et al. (1996) [28]	Seated	Elbow 30°, Wrist 35° ext	Forearm	Distal metacarpals	BREAK	0.86 (Chatillon), 0.81 (MicroFet)	0.55 (Chatillon), 0.52 (MicroFet)
Chou et al. (2008) [29]	Seated	Shoulder 0°, Elbow 90°	/	Dorsal metacarpal region	/	/	/
Nyström Eek et al. (2006) [9]	Supine	Shoulder add, Elbow 0°, Fingers ext	Forearm	Dorsal hand	MAKE	/	/
Escobar et al. (2017) [30]	Supine	Wrist 30° ext, Fingers ext	/	Proximal metacarpophalangeal	BREAK	/	/
Van der Ploeg et al. (1984) [11]	Seated	Wrist neutral	/	Dorsal metacarpal zone	/	/	/
Bohannon (1986) [21]	Supine	Shoulder neutral, Elbow 90°, Wrist neutral, Fingers neutral	Distal forearm	Proximal metacarpophalangeal	MAKE	/	/
Andrews et al. (1996) [33]	Supine	Shoulder neutral, Elbow 90°, Wrist neutral, Fingers neutral	Distal forearm	Proximal metacarpophalangeal	MAKE	/	/
Beenakker et al. (2001) [7]	Seated	Wrist neutral, Fingers flex	/	Proximal third metacarpal head	BREAK	/	/
Bohannon (1996) [38]	/	Shoulder neutral, Elbow 90°, Wrist neutral	/	Proximal metacarpophalangeal	MAKE	/	/
Grooten et al. (2010) [35]	Seated	Shoulder neutral, Elbow 90°, Wrist neutral, Fingers flex	Forearm	Dorsal metacarpophalangeal zone	BREAK	>0.90	0.92
Karahan et al. (2017) [39]	Seated	Elbow semi-flexed, Fingers flex	Forearm fixed to support surface	Dorsal metacarpophalangeal surface	/	/	/
Toemen et al. (2011) [40]	Seated	Shoulder 0°, Elbow 90°, Wrist 0°	Forearm fixed to surface	Distal and dorsal third metacarpal	MAKE	0.91, 0.86	/
Dekkers et al. (2020) [41]	Seated	Elbow 90°, Wrist 0°	/	/	MAKE	/	/
Bohannon et al. (1986) [21]	Seated	Shoulder 0°, Elbow 90°, Wrist neutral	Arm and forearm	Proximal metacarpophalangeal	MAKE	/	/

Legend: /, data not available or not applicable; BREAK, test type where the examiner applies force against the subject until maximal resistance is overcome; ext, extension; flex, flexion; HHD, Handheld Dynamometer; ICC, Intraclass Correlation Coefficient; MAKE, test type where the examiner holds the dynamometer static against the subject’s maximal effort; abd, Abduction; add, Adduction.

This table represents a comprehensive overview of study protocols detailing patient positioning, stabilization of segments, application points for HHD counterforce, types of tests performed, and intra- and inter-examiner reliability (ICC) metrics across various studies involving wrist strength measurement.

### 5.1. Pronation–Supination

Test positions were highly variable in regard to the measurement of forearm pronation and supination: in one study, the test was performed from a supine position with stabilization at the arm level, using a rod that was grasped at one end by the subject, while the dynamometer was applied at the other end. Both the diameter of the rod (2.7 cm), and the position of the dynamometer (20 cm from the center of the hand) was explicitly stated. Conversely, in the second study, the test was conducted in a seated position with stabilization on the proximal forearm, and the counterforce was applied through the dynamometer being positioned 5 cm from the distal epiphysis of the radius on the volar aspect of the forearm (for pronation) or dorsal aspect (for supination).

**Table 4:** Study protocols and reliability assessments for pronation and supination measurements using handheld dynamometers.

Study	Patient Position	Adjacent Segment Position	Stabilized Segment	HHD Counterforce Position	Test Type	*ICC Intra-Examiner*	*ICC Inter-Examiner*
Puharic et al. (1993) [31]	Supine	Shoulder 0°, Elbow 90°, Forearm in intermediate position	Arm	Rod (2.7 cm diameter) held by subject, 20 cm from the center of the hand	/	Pron-sup > 0.92	Pron-sup > 0.90
Toemen et al. (2011) [40]	Seated	Shoulder 0°, Elbow 90°, Forearm in intermediate position, Wrist 0°	Distal to elbow	Volar surface of the wrist, 5 cm from the distal dorsal portion of the radius	MAKE	0.87	>0.81

Legend: ICC, Intraclass Correlation Coefficient; MAKE, type of test where the examiner holds the dynamometer static against the subject’s maximal effort; pron-sup, pronation–supination; /, data not available or not applicable.

This table summarizes the key details of each study’s setup for testing pronation and supination, including patient positioning, the placement and stabilization of segments, dynamometer counterforce positions, type of test conducted, and the reliability metrics (ICC values).

**Table 5:** Protocols and reliability metrics for pronation and supination tests using handheld dynamometers.

Study	Patient Position	Adjacent Segment Position	Stabilized Segment	HHD Counterforce Position	Test Type	*ICC Intra-Examiner*	*ICC Inter-Examiner*
Puharic et al. (1993) [31]	Supine	Shoulder 0°, Elbow 90°, Forearm in intermediate position	Arm	Rod (2.7 cm diameter) held by subject, 20 cm from the center of the hand	/	Pron-sup > 0.92	Pron-sup > 0.90
Toemen et al. (2011) [40]	Seated	Shoulder 0°, Elbow 90°, Forearm in intermediate position, Wrist 0°	Distal to elbow	Dorsal surface of the wrist, 5 cm from the distal dorsal portion of the radius	MAKE	0.94	>0.89

Legend: ICC, Intraclass Correlation Coefficient; MAKE, type of test where the examiner holds the dynamometer static against the subject’s maximal effort; pron-sup, pronation–supination; /, data not available or not applicable.

This table presents the methodologies and reliability metrics for the measurement of pronation and supination using handheld dynamometers, detailing each study’s setup and outcomes.

### 5.2. Radial and Ulnar Deviation

The subject was positioned supine with the forearm pronated and the wrist in a neutral position when radial and ulnar deviation muscle test strength was evaluated; stabilization was applied at the forearm level, while the HHD was applied on the radial edge of the second metacarpal for radial deviation movement and conversely on the ulnar edge of the fifth metacarpal for ulnar deviation. However, it was not possible to compare the procedures as these movement directions were tested in only one study.

**Table 6:** Measurement protocol and reliability for radial deviation using handheld dynamometers.

Study	Patient Position	Adjacent Segment Position	Stabilized Segment	HHD Counterforce Position	Test Type	*ICC Intra-Examiner*	*ICC Inter-Examiner*
Toemen et al. (2011) [40]	Seated	Shoulder 0°, Elbow 90°, Forearm pronated, Wrist 0°	Forearm	Radial edge of the second metacarpal	MAKE	0.71	>0.87

This table details the test setup for radial deviation measurements by Toemen et al., highlighting the positioning of the patient and the handheld dynamometer, as well as the intra- and inter-examiner reliability metrics.

**Table 7:** Measurement protocol and reliability for ulnar deviation using handheld dynamometers.

Study	Patient Position	Adjacent Segment Position	Stabilized Segment	HHD Counterforce Position	Test Type	*ICC Intra-Examiner*	*ICC Inter-Examiner*
Toemen et al. (2011) [40]	Seated	Shoulder 0°, Elbow 90°, Forearm pronated, Wrist 0°	Forearm	Ulnar edge of the fifth metacarpal	MAKE	0.90	>0.77

This table provides details on the ulnar deviation measurement protocol used by Toemen et al., including the stabilization and positioning of the handheld dynamometer, as well as intra- and inter-examiner reliability metrics.

### 5.3. Types of Tests Used 

With an HHD, two types of tests are possible: the “make test,” where the examiner holds the dynamometer in a static position on the subject’s segment while the subject pushes, exerting maximum force against the instrument, or the “break test,” where the examiner pushes the dynamometer against the segment to be tested until the subject’s maximum force is overcome and the joint yields, moving in the opposite direction. Although the literature does not support the theory that one test is superior to the other, some authors argue that the two modes test different aspects of muscle strength. According to a study by Bohannon (conducted on elbow flexors), the “make test” appears to evaluate the concentric component more, while the break test involves a minimal part of eccentric muscle contraction, which is known to lead to higher strength values compared to concentric contraction alone.

### 5.4. Types of HHDs

From the analysis of the included studies, it appears that the devices used for assessments were mostly different models of dynamometers (Table 8). These are always “hand-held” dynamometers, but they can vary in shape and intrinsic characteristics of the instrument. Listed below, are all the different instruments that have been used for the assessment of the wrist in the selected studies. A complete overview of the handheld dynamometers used in the included studies is provided in Table 8 and Table 9.

**Table 8:** Overview of handheld dynamometer models and their utilization in research studies.

Device Model	Manufacturer	Number of Articles
Model 01165	Lafayette Instrument, Lafayette, IN, USA	2
Model 01163	Lafayette Instrument, Lafayette, IN, USA	1
NMMT model 01160	Lafayette Instrument, Lafayette, IN, USA	2
(Not Specified)	Lafayette Instrument, Lafayette, IN, USA	1
MicroFet2 TM	Hoggan Scientific, Salt Lake City, UT, USA	3
MicroFet	Hoggan Scientific, Salt Lake City, UT, USA	2
CSD500	Chatillon Medical Products, Greensboro, NC, USA	1
CSD400C	Chatillon, Agawam, MA, USA	1
(Not Specified)	John Chatillon & Sons, Kew Gardens, NY, USA	2
Accuforce II	Ametek, Largo, FL, USA	3
Spark	SPARK Instruments & Academics Inc., Coralville, IA, USA	1
Strain-Gauge-Type Compact Force Sensor LPR-A1KNS1	Kyowa Electronic Instruments Co., Tokyo, Japan	1
Modified Pressure Gauge	Wika Group, Jebel Ali, Dubai	1
CT 3001	C.I.T. Technics, Groningen, The Netherlands	1
JTech Commander PowerTrack II	JTECH Medical, Salt Lake City, UT, USA	1

This table lists the different models of handheld dynamometers used in various studies, detailing their manufacturers and the number of articles in which each model was featured.

In 1996, Kimura et al. began to address the issue of using HHD instruments with different characteristics. In a study comparing the reliability of the HHD across specific models—MicroFet and Chatillon—they found that the force values recorded through the two models were not interchangeable, concluding that there was not sufficiently high reliability in measurements made on the same subject with different devices.

More recently, the question was revisited in a study focusing on the use of the Nicholas Manual Muscle Tester (NMMT) exclusively for wrist extension movement. This study did not compare different models but noted that the reliability of measurements reported was specific to the NMMT alone, and further investigation into inter-device reliability was necessary. Currently, no other studies in the context of evaluating the wrist in healthy subjects have highlighted the criticality of inter-device reliability.

Bringing this issue back to clinical practice, it becomes clear that the same instrument should always be used for evaluations and, at the same time, if a comparison is made between the measured values in the patient and the normative values reported in the literature, it should be considered which instrument recorded these normative values.

Although all instruments used were hand-held dynamometers, their extrinsic and intrinsic features varied. These included ergonomic shapes, surface interfaces for contact, calibration abilities, and measurement range. Some authors [37] investigated the impact of inter-device variability, reporting low reliability when comparing measurements across different HHD models. This supports the clinical recommendation of consistently using the same HHD for both assessment and comparison with normative data.

### 5.5. Reliability of Dynamometric Tests

Analyzing the data on intra- and inter-operator reliability, it is evident that in most cases, the values expressed fall within the “high” range (ICC between 0.71 and 0.90). Intra-operator reliability is documented in several studies within this range, fluctuating from a minimum ICC value of 0.71 to a maximum of 0.95. On the other hand, inter-operator reliability, in some cases, shows values that do not fall within the high reliability range. However, there are studies (McMahon ′92, Kimura ′96) that present ICC values of 0.32, 0.52, and 0.55. but besides these two studies, other studies have reported ICC values for inter-operator reliability ranging from 0.77 to 0.93. The data thus support the hypothesis of greater reliability of intra-operator measurements compared to inter-operator measurements, consistent with the plausible presence of measurement bias related to the characteristics of the subjects involved or presumably also to a lack of precise and shared standardization of position.

### 5.6. Normative Values

Some of the studies analyzed have attempted to identify normative values for wrist force evaluation using a handheld dynamometer. One study identified normative values for the adult female population and another for the general adult population. The values vary based on different measurement units used and in relation to different aspects considered in the individual study, such as age, gender, and dominant arm (Table 9).

**Table 9:** Normative force values for wrist extension using handheld dynamometers across different age groups and genders.

Study	HHD	Tested Movements	Number of Subjects Tested	Age Range	Normal Force Values (Dominant Arm, Female)	Normal Force Values (Non-Dominant arm, Female)	Normal Force Values (Dominant Arm, Male)	Normal Force Values (Non-Dominant Arm, Male)
Bohannon (1996) [32]	Accuforce II	Extension (E)	123	20–29, 30–39, 40–49, 50–59, 60–69, 70–79	Min 69.8 N, Max 104.6 N	Min 61.4 N, Max 104.6 N	N/A	N/A
Andrews et al. (1996) [33]	Chatillon CSD400C	Extension (E)	156	50–59, 60–69, 70–79	Min 79.0 N, Max 90.8 N	Min 70.5 N, Max 82.8 N	Min 124.9 N, Max 149.1 N	Min 119.9 N, Max 139.2 N

This table provides a detailed view of the studies examining wrist extension force, detailing the dynamometer used, the number of subjects tested, their age range, and the normative values of force for both dominant and non-dominant arms, separated by gender where applicable.

## 6. Discussion

**Figure 2:**
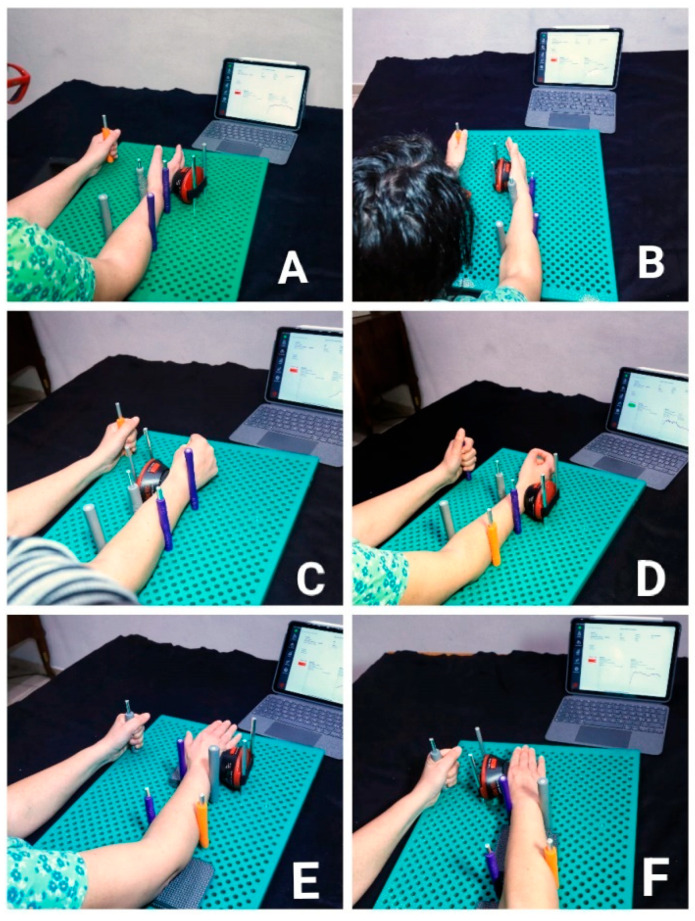
Pegboard joint stabilization table (PJST). Sequential demonstration of a standardized protocol for wrist strength assessment using a handheld dynamometer. Each panel illustrates the testing setup and various stages of the assessment process: (**A**) initial setup with the patient positioned for the test; (**B**) examiner aligning the dynamometer on the patient’s wrist; (**C**) measurement of wrist flexion strength; (**D**) measurement of wrist extension strength; (**E**) measurement of radial deviation strength; (**F**) measurement of ulnar deviation strength. This sequence ensures consistent positioning and device application for accurate and reliable strength measurement.

In the field of physical therapy and orthopedics, handheld dynamometry (HHD) is extensively used for assessing muscle strength, particularly in the wrist [2,17,18]. However, the reliability of these measurements can vary significantly depending on several factors including intra-examiner and inter-examiner consistency. Intra-examiner reliability is crucial as it ensures that the same examiner can produce consistent results across multiple tests under identical conditions, making it indispensable in monitoring the progression or regression of a patient’s muscle strength over time [9,31,38]. On the other hand, inter-examiner reliability reflects the degree to which different examiners can replicate results under the same testing conditions. This form of reliability often reveals variability, as highlighted by research including a notable study by Kimura et al. [28], which found discrepancies in force measurements when different examiners conducted the same tests using the same HHD models. These findings suggest inherent methodological challenges and highlight the significant impact of individual examiner techniques and physical characteristics. The methodological variables affecting HHD measurements are manifold. Patient positioning, the precise application point of the HHD on the tested segment, and the choice of test method (the Make or Break test) are all critical in determining the accuracy and reliability of the results. The “Make” test, where the examiner applies a static force against the subject’s maximal effort, tends to yield more reproducible results. In contrast, the “Break” test, which measures the force as the examiner overcomes the subject’s effort, introduces variables like eccentric muscle contraction, known to produce higher force values but also greater variability. Standardizing the testing procedure is vital in overcoming these challenges [4,7]. A more proximal application point of the dynamometer on the tested segment can increase measurement variability, especially in small segments such as the wrist [4,7,10,11,12]. Each muscle follows a length–tension relationship; therefore, depending on whether the muscle is shortened or lengthened during testing, the maximal force production may vary [14]. For instance, adjustments in the dynamometer’s position relative to the joint being tested have been shown to influence the recorded force significantly. Studies have indicated that positioning the dynamometer closer to the joint axis often results in higher force readings but requires greater stabilization effort by the examiner, thus introducing potential variability. In the case of wrist assessments, even minor deviations in dynamometer placement can lead to substantial differences in force measurements due to the small size and complex biomechanics of the wrist and hand [29,40,42]. To address these issues, our proposed protocol includes the use of a standardized setup with a specifically designed Pegboard Joint Stabilization Table (PJST) (Figure 2) that provides a stable platform for both the subject’s limb and the HHD. While mechanical stabilization may reduce inter-examiner variability, patient-related biases such as fatigue, anxiety, or lack of understanding of the test procedure could still affect the measurements. Future protocols should aim to minimize these through patient education and pre-test familiarization. This table facilitates precise positioning and stabilization, ensuring that the dynamometer’s application point and the subject’s limb position are consistently maintained throughout tests. This approach is particularly beneficial when assessing smaller muscle groups such as those in the wrist, where precise measurement is crucial. Moreover, the proposed standardized procedure includes detailed guidelines for the positioning of the patient and the HHD, ensuring that the forearm, wrist, and hand are correctly aligned and supported to prevent any undue movement or variability during testing. For example, the protocol specifies the degree of pronation or supination of the forearm, the wrist’s angle of flexion or extension, and the exact placement of the dynamometer relative to specific anatomical landmarks. While handheld dynamometry is a valuable tool for assessing muscle strength, its efficacy is heavily dependent on the standardization of testing procedures. The introduction of a robust standardized protocol, as suggested, could significantly enhance the reliability and validity of HHD measurements, providing clinicians and researchers with more accurate and consistent data. This would not only improve patient care by enabling the more precise monitoring of recovery and treatment effects but also facilitate more reliable research outcomes that could inform future clinical practices and interventions.

The following protocol proposals were developed considering all the potential measurement biases identified in the literature:

- **Flexion/Extension test**: Seated position with shoulder at 30° abduction, elbow at 90°, forearm neutral and stabilized with four pegs. Wrist and fingers in neutral. The HHD is fixed to the test table, and the contact point is on the distal metacarpal. The test is performed using the “MAKE” method.
- **Ulnar/Radial Deviation**: Same seated setup but with the forearm in pronation. Foam pads may be used to centralize HHD contact.
- **Pronation/Supination**: Similar setup; HHD is applied on the volar side of the distal radius for pronation and on the dorsal side for supination. Stabilization is achieved with pegs and support pads.

While the enhancements proposed for using handheld dynamometry in wrist strength assessment offer a robust framework, this study is not without limitations. The scope of data is confined predominantly to wrist assessments, potentially limiting the applicability of findings to other body parts or complex, multi-joint movements. The variability inherent in the methodologies, populations, and equipment used across the analyzed studies introduces a level of inconsistency that could affect the synthesis of data and making broad conclusions. Additionally, our review relies heavily on available published studies, which may not represent the entire spectrum of research in this area, particularly by excluding unpublished studies and ongoing research that could provide further insights. These limitations underscore the need for cautious interpretation of the findings and suggest that further research is necessary to confirm these results and expand the applicability of the proposed standardized protocol for handheld dynamometry.

## 7. Clinical Practice Implications

The implications for clinical practice from our study on handheld dynamometry in wrist strength assessment are significant yet need careful consideration due to the inherent limitations of the review. Properly implementing the standardized protocols can lead to more accurate and consistent measurements of muscle strength, crucial in monitoring rehabilitation progress and treatment outcomes. This can enhance the precision of interventions and ensure better patient care by allowing clinicians to tailor treatments [29,35,40] based on reliable data. Moreover, standardization reduces variability in results caused by different examiners and testing conditions, contributing to more dependable assessments across different clinical settings. This could ultimately facilitate better clinical decision-making and improve outcomes for patients undergoing recovery from wrist injuries or disorders.

## 8. Conclusions

The review of handheld dynamometry for wrist strength assessment highlights the potential for improved measurement reliability through standardized testing protocols. Implementing these standardized methods can significantly enhance clinical assessments, providing clinicians with accurate tools to monitor rehabilitation progress. However, the variability in methods and dependence on published data lead us to caution against overgeneralizing the findings. Further research and a broader adoption of uniform testing procedures are recommended to solidify these protocols as standards in clinical practice, thereby enhancing the overall quality of patient care and treatment efficacy.
